# The forbidden doubling: exploring rare spermatocyte polyploidy in mammals

**DOI:** 10.3897/compcytogen.20.177662

**Published:** 2026-01-23

**Authors:** Sergey Matveevsky, Oxana Kolomiets, Tatiana Grishaeva, Aleksey Bogdanov, Valentina Tambovtseva, Irina Bakloushinskaya

**Affiliations:** 1 Vavilov Institute of General Genetics, Russian Academy of Sciences, Moscow 119991, Russia Vavilov Institute of General Genetics, Russian Academy of Sciences Moscow Russia; 2 Koltzov Institute of Developmental Biology, Russian Academy of Sciences, Moscow 119334, Russia Koltzov Institute of Developmental Biology, Russian Academy of Sciences Moscow Russia

**Keywords:** Gametogenesis, meiotic prophase I, tetraploidy, quadrivalent, synaptonemal complex

## Abstract

We first studied several rare cases of over-diploid spermatocyte emergence using advanced immunocytochemical methods and a cross-species approach in subterranean rodents *Ellobius tancrei* (Blasius, 1884), *E. alaicus* Vorontsov et al., 1969, *E. talpinus* (Pallas, 1770), and *Nannospalax leucodon* (Nordmann, 1840) (all belong to the order Rodentia). The tetraploid spermatocytes exhibited specific features during meiotic prophase I, including symmetric and asymmetric chromosome quadrivalents with partner-switching, extended asynapsis, altered recombination patterns, and variable chromatin inactivation. These anomalies suggest that meiotic checkpoints, which are potentially triggered by failed synapsis or incomplete sex chromosome silencing, may act to prevent progression of polyploid spermatocytes. However, the quadrivalents assembled shelterin complexes at chromosome ends, as observed in *E. talpinus*, and these ends were connected to the nuclear envelope through the linker of nucleoskeleton and cytoskeleton (LINC) complex, as observed in *E. alaicus*, similarly to normal spermatocytes.

## Introduction

Polyploidy, i.e., the presence of more than two complete chromosome sets in some or all cells of an organism, is widespread among eukaryotes and contributes to evolutionary diversification. In plants, it represents a central and recurrent driver of speciation, while in animals its evolutionary impact is mainly observed in invertebrates and lower vertebrates. Polyploidy in somatic cells of the liver, pancreas, heart, and muscles has been well documented in animals and humans (Fox and Duronio 2013; Wang et al. 2017). Certain tissues in mammalian females, such as the placenta and lactating mammary glands, also exhibit polyploidy, which may enhance the organ function (Zybina and Zybina 2020; Anatskaya and Vinogradov 2022).

Despite numerous cases of somatic polyploidy, whole-genome duplication is strongly constrained in mammals. In contrast to plants, where polyploidy frequently drives speciation, polyploid cells in animals are often eliminated through apoptosis and fail to contribute to gametogenesis. In mammals, the presence of heteromorphic sex chromosomes has been proposed as a key factor preventing the establishment of polyploidy (Ohno 1970; Stöck et al. 2021), providing a mechanistic explanation for the so-called “forbidden doubling”. In addition, the increased nuclear volume and chromosome number in polyploid cells may compromise nuclear architecture, interfere with chromosome interactions, and impair mitotic segregation (Shu et al. 2018; Vittoria et al. 2023). Finally, polyploidy is exceptionally rare in the mammalian germline and is generally regarded as a deviation from normal haploid gamete production, as polyploidy during gametogenesis can potentially lead to aneuploid progeny (Mason and Pires 2015). Furthermore, although diploid gametes may be formed through abnormal mitotic divisions, such as endoreduplication or failed cytokinesis (Therman and Susman 1993; Egozcue et al. 2000; Escalier 2002; Lee et al. 2009; Zielke et al. 2013; Stenberg and Saura 2013), these cells are typically eliminated by meiotic checkpoints, especially in males (Li et al. 2009). In contrast, female meiosis appears to be more error-prone, and diploid oocytes are more likely to contribute to over-diploid embryos in humans (Beatty and Fischberg 1952; Speed 1985; Capalbo et al. 2017; Samura et al. 2023).

There are only a few reports of polyploid spermatocytes in model mammals (Pogosianz and Brujako 1971; Chandley et al. 1974; Holm and Rasmussen 1983) and in humans (Carothers and Beatty 1975; Kajii and Niikawa 1977; Wegner et al. 2001; Sarrate et al. 2014; Pearson and Madan 2018). Although sporadic instances of meiotic polyploidy have been reported in mammals, cytologically characterized cases of autotetraploid spermatocytes, particularly those with fully assembled synaptonemal complexes (SCs), remain exceedingly rare. To date, only isolated cases have been documented in mice (Solari and Moses 1977), humans (Codina-Pascual et al. 2006; Schmidt 2017), northern mole vole (Matveevsky et al. 2017), and, recently, in brown bears (Virabyan et al. 2025), leaving major gaps in our understanding of how polyploid germ cells behave during meiosis and how they are regulated. The mechanisms, by which tetraploid meiocytes progress through early prophase I, resolve complex multivalent configurations, and interact with meiotic surveillance systems remain largely unknown.

In this study, we aimed to identify and characterize rare tetraploid spermatocytes in selected rodent species, including mole voles and mole rats, using immunocytochemistry. We focused on the structural configurations of the SCs, recombination, and transcriptional silencing of chromatin during meiotic prophase I. By comparing these features with those observed in diploid cells, we sought to provide cytological insights into the fate of naturally occurring polyploid spermatocytes and to explore the potential involvement of meiotic checkpoints that may eliminate or arrest cells carrying unresolved multivalents. By integrating comparative cytogenetic data with functional markers of telomere attachment (RAP1) and nuclear envelope interactions (SUN1), our study was aimed at elucidation of how mammalian meiosis restricts polyploid germ cell formation and maintains genomic stability.

## Material and methods

In total, 12 polyploid spermatocytes were studied from six males of four rodent species (order Rodentia). One lesser blind mole rat *Nannospalax leucodon* (Nordmann, 1840) (2n = 56, ID #2019-01) was captured near Ravno Pole village, eastern Sofia, Bulgaria (Matveevsky et al. 2021). Two males of the eastern mole vole *Ellobius tancrei* Blasius, 1884 were obtained through prolonged experimental breeding of distinct chromosome forms (2n = 49, F2, ID #25187 and 2n = 49, F10, ID #27430, see Kolomiets et al. 2023). One male of the Alay mole vole *Ellobius alaicus* Vorontsov et al., 1969 (2n = 52, ID #27495) from southeastern Kyrgyzstan (see Tambovtseva et al. 2022) and two males (2n = 54, IDs #27416 and #27041) of the common (northern) mole vole *E. talpinus* (Pallas, 1770), offspring of experimental breeding, were provided by the Large-Scale Research Facility “Collection of Wildlife Tissues for Genetic Research,” IDB RAS, registration no. 3579666. All procedures followed institutional and international ethical standards (Ethics Committee approvals: VIGG RAS Order No. 3, 10 November 2016; IDB RAS Protocol No. 37, 25 June 2020).

Preparations of meiotic chromosomes at the stage of diakinesis and metaphase I regularly reveal cells resembling tetraploid ones, but it is difficult to confirm that these cells are truly polyploid because of their possible overlapping. The best way to confirm genuine tetraploidy of spermatocytes is to identify nuclei in the middle of prophase I, when chromosome synapsis occurs. Cell spreads for studying synaptonemal complex (SC) were prepared following Peters et al. (1997); some details see Matveevsky et al. 2021.

The following primary antibodies were used: anti-SYCP3 (synaptonemal complex protein 3; rabbit, 1:250, #15093, Abcam, UK) to label axial/lateral elements of SCs; anti-centromere (CREST) (human, 1:250, #90C, Fitzgerald, USA) to identify centromeres (kinetochore proteins); anti-γH2AFX (phosphorylated form of the H2A histone family member X (H2AX); mouse, 1:250–500, #22551, Abcam) to detect chromatin inactivation; anti-MLH1 (MutL Homolog 1; mouse, 1:50, #14206 Abcam) as a recombination marker; anti-RAP1 (Repressor/Activator Protein 1; rabbit, 1:100, #NB100-56321, NovusBio) to detect telomere sites; anti-SUN1 (Sad1 and UNC-84 domain–containing protein 1; rabbit, 1:250, #ab74758, Abcam) to visualize components of nuclear envelope, LINC (Linker of Nucleoskeleton and Cytoskeleton). Secondary antibodies included goat anti-rabbit IgG Alexa Fluor 488, anti-human IgG Alexa Fluor 546, and anti-mouse IgG Alexa Fluor 546/555 (Invitrogen, USA; dilution 1:300–800). Slides were washed in PBS and mounted with Vectashield containing DAPI (4',6-diamidino-2-phenylindole; Vector Laboratories, USA). Visualization was performed using an Axio Imager D1 fluorescence microscope (Carl Zeiss, Germany). The immunostaining protocols followed previously described procedures (Matveevsky et al. 2021).

A “quadrivalent” designation was used in this paper to characterize SC configurations consisting of four axes in tetraploid meiocytes, in contrast to a “tetravalent” term usually used for SC configurations in chromosomally heterozygous (hybrid) organisms, such as in our previously published work (Matveevsky et al. 2015).

## Results

Meiotic spreads from *E. tancrei*, *E. alaicus*, *E. talpinus*, and *N. leucodon* specimens revealed the presence of several tetraploid spermatocytes. These cells were confidently distinguished from overlapping diploid cells through careful analysis of the synapsis patterns during prophase I combined with the application of multiple immunocytochemical markers targeting key meiotic proteins. The use of these markers allowed for the visualization of synaptonemal complex formation, centromere positioning, and DNA damage signalling, providing robust criteria for the identification of true tetraploid cells. This approach ensured that the observed tetraploid configurations reflected genuine polyploidy rather than artefacts of cell overlap.

### Case Study 1: *Ellobius tancrei*

**Figure 1.**
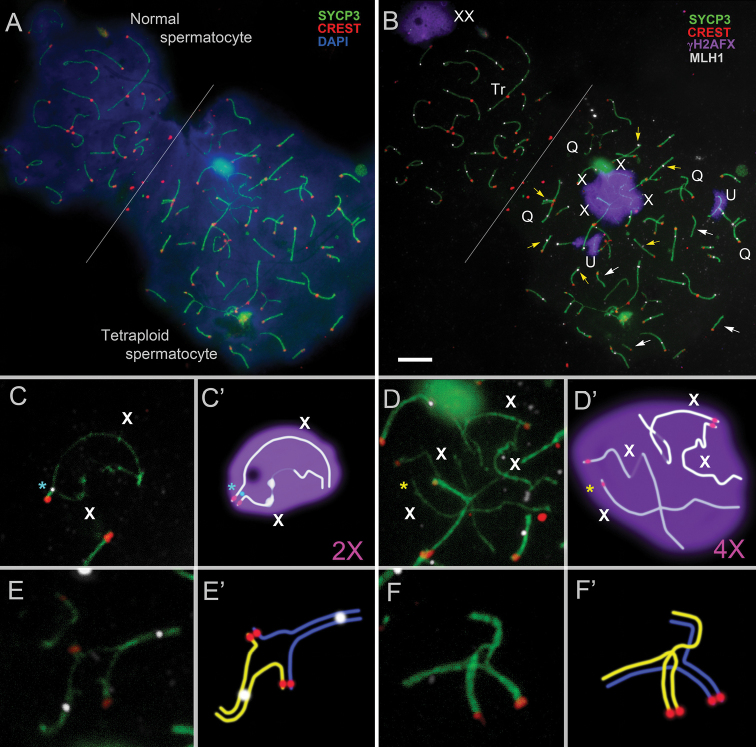
Normal (diploid) and tetraploid pachytene spermatocytes of *E. tancrei* #27430, 2n = 49, 2Rb2.18, 1Rb4.12, 2Rb9.13. Axial and lateral elements of the SCs were identified using an anti-SYCP3 antibody (green); centromeres were identified using “CREST”—antibodies to kinetochore proteins (red); anti-MLH1 antibodies (white) were used as a marker of recombination nodules; and chromatin inactivation was revealed using an anti-γH2AFX antibody (violet). Chromatin was stained with DAPI (blue). Fragments of the microscopic image (**C–F**) were added by corresponding interpretation schemes (**C’–F’**). X – sex chromosomes, Q – chromosomal quadrivalent, Tr – chromosomal trivalent, U – univalent. Both spermatocytes were photographed in one microscopic image without changes in their position. Normal and tetraploid spermatocytes differ from each other in cell size (see DAPI in **A**) and the presence of quadrivalents in the latter (see Q in **B**). In the normal (diploid) spermatocyte, 24 centromeric foci and 28 MLH1 foci are observed, whereas in the tetraploid spermatocyte, 57 centromeric foci and 39 MLH1 foci are present. White arrows point to SC bivalents without MLH1 dots (**B**). Yellow arrows indicate SC bivalents with shifted MLH1 foci (**B**). A blue asterisk indicates the presence of a recombination nodule in the sex (2X) bivalent in the normal spermatocyte (**C, C’**). In the tetraploid spermatocyte, four axes, which corresponded to four X chromosomes (4X), were observed within one γH2AFX cloud (**B, D, D’**); the centromeric region is not visualized on one of the X axes (yellow asterisk). One quadrivalent has two MLH1 dots (**E, E’**), another quadrivalent is without MLH1 signals (**F, F’**). Scale bar: 5 µm.

Out of the thousands of spermatocytes analyzed during the last dozen years from males of chromosomally variable species *E. tancrei* represented by different karyotypic forms, only two cells, which were obtained from experimental hybrids, were unambiguously identified as tetraploid (Fig. 1, Suppl. material 1: figs S1A, S2). Specimen #27430 (2n = 49, 2Rb2.18, 1Rb4.12, 2Rb9.13) was heterozygous for one Robertsonian translocation and sterile as it was previously shown (Kolomiets et al. 2023). The single tetraploid cell from this individual exhibited four SC quadrivalents, two univalents, and several trivalent-like configurations. Four X chromosome axes, two of which were fragmented, were located within a γH2AFX-positive part of chromatin (Fig. 1). The analysis of MLH1 signals revealed the presence of 39 recombination foci in the cell. This value is substantially higher than the average for diploid cells (21.6) and exceeds the maximum number of recombination foci that have been previously observed in diploid pachytene spermatocytes (34 dots; see Kolomiets et al. 2023). The presence of autosomal quadrivalents as well as a quasi-tetravalent configuration of the four X chromosomes separated by a γH2AFX-positive chromatin cloud, together with the increased total number of MLH1 foci, refutes the assumption that this pattern represents an overlay of two diploid cells and instead confirms tetraploid nature of this spermatocyte. Although some bivalents lacked MLH1 foci or showed shifting and atypical localization of the latters, the overall elevated number of the foci reflects a re-patterning of the recombination landscape consistent with tetraploidy. Thus, quantification of MLH1 foci serves as an informative complementary parameter for the analysis of quadrivalent chromosome configurations and centromere counts, enabling a more comprehensive assessment of meiotic events in tetraploid spermatocytes.

A single tetraploid diplotene cell (Suppl. material 1: fig. S2) was found in male #25187 (2n = 49, 1Rb2.11, 1Rb2.18, 2Rb5.9, 1Rb3.18), also sterile. The presence of multivalents due to Robertsonian translocations and univalents compromises the normal completion of meiotic prophase I and probably leads to elimination of the cell.

### Case Study 2: *Ellobius alaicus*

Two tetraploid nuclei were identified in one specimen of *E. alaicus*. SC quadrivalents showed symmetrical configurations with asynapsis in proximal regions (Suppl. material 1: figs S3, S4). SUN1 immunostaining revealed chromosomal end-attachment to the nuclear envelope, implicating the LINC complex involvement in SC architecture. Sex chromosomes could not be identified because of insufficient morphological and immunocytochemical specificity.

### Case Study 3: *Ellobius talpinus*

**Figure 2.**
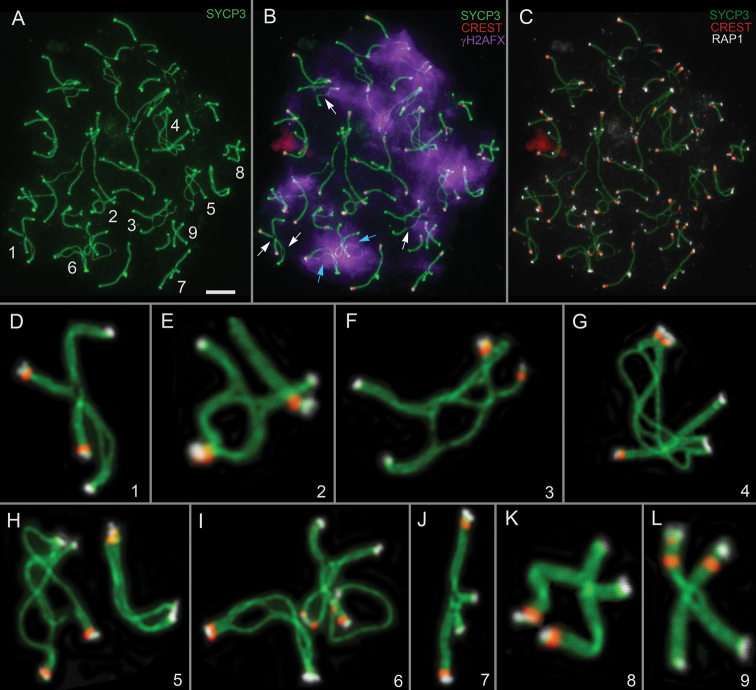
Tetraploid pachytene spermatocyte of common mole vole *E. talpinus*, #27416 (**A–C**). Axial and lateral elements of SCs were identified using an anti-SYCP3 antibody (green); centromeres were identified using “CREST”—antibodies to kinetochore proteins (red); telomeres were detected using anti-RAP1 antibody (white); and chromatin inactivation was revealed using an anti-γH2AFX antibody (violet). Enlarged quadrivalents are shown in panels **D–L**. Cyan arrows indicate γH2AFX-positive asynaptic areas. White arrows indicate γH2AFX-negative chromosomal regions. Scale bar: 5 µm.

Spermatocytes, exhibiting features of tetraploid cells, were identified in two males (#27416 and #27041) of the common mole vole *E. talpinus*. In one of such pachytene spermatocytes (#27416 specimen), both symmetric and asymmetric quadrivalents with and without partner switching were visible (Fig. 2A–L, Suppl. material 1: fig. S1B). We previously found similar quadrivalents in this species (Matveevsky et al. 2017). Immunodetection of the RAP1 protein revealed for the first time that both quadrivalents and resolved bivalents, which had escaped from their quadrivalent configurations, formed shelterin complexes in telomeric regions (Fig. 2C–L). Of particular interest is the finding that both γH2AFX-positive and γH2AFX-negative axes were identified as part of the quadrivalents in this cell (see white and cyan arrows in Fig. 2B). Sex chromosomes could not be reliably distinguished due to lack of morphological and immunocytochemical markers, as was also the case in a zygotene spermatocyte with quadrivalent-like configurations (#27041 specimen, Suppl. material 1: fig. S5).

### Case Study 4: *Nannospalax leucodon*

**Figure 3.**
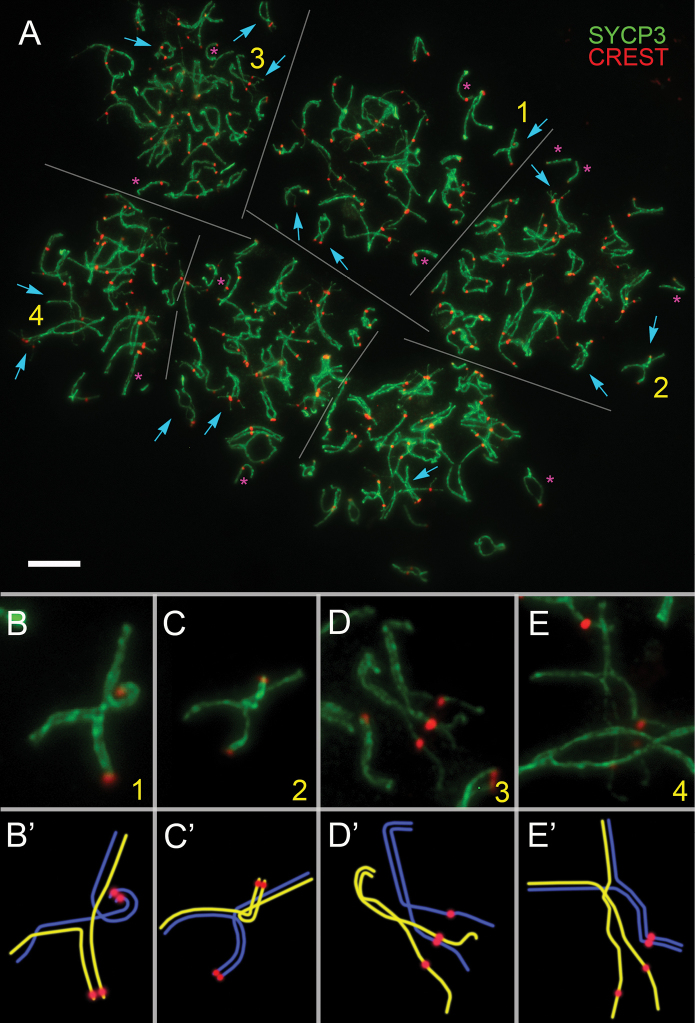
Six tetraploid pachytene spermatocytes of the lesser blind mole rat *N. leucodon* (**A**). Axial and lateral elements of SCs were identified using the anti-SYCP3 antibodies (green); centromeres were identified using “CREST”—antibodies to kinetochore proteins (red). Yellow numbers indicate those parts of the nuclei that are enlarged in insets (**B–E**) added also by corresponding interpretation schemes (**B’–E’**). Cyan arrows point to SC quadrivalents and quadrivalent-like SC configurations. Magenta asterisks indicate separate SC bivalents. Scale bar: 5 µm (**A**).

In the 56-chromosomal form of *N. leucodon*, six tetraploid spermatocytes were found among 251 cells at different pachytene substages (Fig. 3, Suppl. material 1: fig. S1C). In the presented tetraploid spermatocytes of the lesser blind mole rat, the number of centromeres was approximately 2-fold higher (58 instead of 28 in diploids), supporting tetraploidy. Chromosomes appeared as SC bivalents and various quadrivalent-like formations. We were unable to identify sex chromosomes.

Quadrivalents displayed both symmetric (fully synapsed arms) and asymmetric configurations (with incomplete synapsis or desynapsis). Partner-switching within quadrivalents was common, and asynapsis was prominent in certain regions.

## Discussion

The analysis of four rodent species revealed that tetraploid spermatocytes rarely occur and display consistent cytological features. These abnormal cells were characterized by the formation of quadrivalents with partial asynapsis, elevated numbers of MLH1 recombination foci, and distinct abnormalities of the sex chromosomes, including their central localization, fragmentation, and intense γH2AFX staining. Additionally, the SUN1-positive chromosomal ends indicated nuclear envelope anchoring of quadrivalents. Together, these findings demonstrate that while tetraploidy in male rodent meiosis occurs sporadically, it produces structurally unique meiotic configurations that differ fundamentally from normal diploid spermatocytes.

Constitutional polyploidy is characteristic of a large number of plants and lower vertebrates, but there are no such examples for mammals (Muller 1925; Otto and Whitton 2000; Gregory and Mable 2005; Song et al. 2012). In *Tympanoctomys barrerae* (Lawrence, 1941), evidence from GISH on mitotic metaphase cells, together with W-CGH and immunodetection analyses on pachytene-stage meiotic cells, supports allotetraploidy (Gallardo et al. 1999; Suárez-Villota et al. 2012), yet this remains a unique case.

Since the discovery of chromosomes, studies of gametogenesis in polyploid organisms, particularly meiosis, have been a focus of attention (Newton and Darlington 1929; White 1933; McCollum 1957). In this context, the processes of synapsis and chromosome recombination in prophase I of meiosis have been mainly emphasized in plants and occasionally in insects (Rasmussen 1986; Higgins et al. 2014). Despite the fact that cases of sporadic meiotic polyploidy have been identified, autotetraploid meiocytes with SCs are very rarely detected. To our knowledge, including the results of this work, there are only nine studies of SCs in tetraploid spermatocytes to date: two in humans, one in the brown bear, and six in various rodent species (Table 1).

**Table 1.** Synaptonemal complexes in mammalian tetraploid spermatocytes.

**Species (2n**)	**Mouse *M. musculus* (40)**	**Human *H. sapiens* (46)**	**Human *H. sapiens* (46)**	**Brown bear *U. arctos* (74)**	**Mole vole *E. talpinus* (54)**	**Mole vole *E. talpinus* (54)**	**Mole vole *E. tancrei* (49)**	**Mole vole *E. alaicus* (52)**	**Mole rat *N. leucodon* (56)**
**Type of polyploidy**	Autotetraploidy	Autotetraploidy	Autotetraploidy	Autotetraploidy	Autotetraploidy	Autotetraploidy	Autotetraploidy	Autotetraploidy	Autotetraploidy
**Meiotic stage**	Pachytene	Pachytene	Pachytene	Late zygotene /early pachytene	Pachytene	Zygotene and pachytene	Pachytene	Pachytene	Pachytene (various substages)
**Number of tetraploid nuclei**	1 (among hundreds)	1 (among 105)	2 cells found	1 (among 389)	1 cell found (among more than 400 cells)	2 cells found (1 among 142 cells in #27416; 1 among 210 cells in #27041)	1 cell found (among more than 500 cells)	2 cells found (among more than 700 cells)	6 cells found (among 251)
**SC configurations**	6 quadrivalents and 3 quadrivalent-like configurations + 20 bivalents	5 quadrivalents + bivalents	Some quadrivalent-like structures + bivalents	3 quadrivalents + bivalents	3 quadrivalents and 7 quadrivalent-like configurations + bivalents	7 clearly distinguishable quadrivalents and 6 quadrivalent-like configurations + bivalents	4 quadrivalents + 2 univalents + bivalents	10 quadrivalents and 7 quadrivalent-like configurations	2–3 quadrivalents and 1–3 quadrivalent-like configurations per cell + bivalents
**Sex chromosomes**	4 thick sex axes (2X+2Y) (not clear in microphoto)	2 separate bivalents (XX + partial Y-Y synapsis)	Not described	Not described	Not described	Not identified	4X (separate axes, fragmented, centrally located)	Not identified	Not identified
**Recombination (MLH1)**	Not analyzed	73 foci per cell (>diploid but < 2x)	Not analyzed	Not analyzed	Not analyzed	MLH1 foci absent in this cell; present in neighbors	39 foci per cell (>diploid but < 2x)	Not analyzed	Not analyzed
**Switchings of partners in some quadrivalents**	Single and double switchings	Single switchings	Not clear in microphoto	No switchings	Single switchings	Single switchings	Single switchings	Single switchings	Single switchings
**Symmetry of quadrivalents**	Symmetrical quadrivalents	Symmetrical and asymmetrical quadrivalents	Likely asymmetrical quadrivalent-like structures	Symmetrical quadrivalents	Symmetrical quadrivalents	Symmetrical and asymmetrical quadrivalents	Symmetrical quadrivalents	Symmetrical quadrivalents	Symmetrical and asymmetrical quadrivalents
Presumed origin *	Nuclear fusion or tetraploid spermatogonia	Pre-meiotic endoreduplication	Pre-meiotic endoreduplication	Pre-meiotic endoreduplication	Pre-meiotic endoreduplication	Pre-meiotic errors	Pre-meiotic errors	Pre-meiotic errors	Pre-meiotic errors
Possible cell fate *	Likely degeneration	Possible 2n sperm	Possible 2n sperm	No explanation	Possible 2n sperm, likely elimination	Likely elimination	Likely elimination	Likely elimination	Likely elimination
**Methods and Markers**	Electron microscopy (AgNO_3_-staining)	ImmunoCyto (SYCP3, CREST, MLH1) + stM-FISH	ImmunoCyto (SYCP3, CENP-E)	ImmunoCyto (SYCP3, ACA)	ImmunoCyto (SYCP3, CREST, RAD51)	ImmunoCyto (SYCP3, CREST, RAP1, γH2AFX)	ImmunoCyto (SYCP3, CREST, MLH1, γH2AFX)	ImmunoCyto (SYCP3, CREST, SUN1)	ImmunoCyto (SYCP3, CREST)
**Unique features**	First description of quadrivalents in mammals	Chr 1, 2, 9, 16, 20 each form a quadrivalent	Patient-derived cells (obstructive azoospermia)	First meiotic description in bears	All stages (1–4) of quadrivalent resolution	Shelterin (e.g., RAP1) at telomeres in quadrivalents	Quadrivalents with/without recombination nodules	SUN1-mediated NE attachment of quadrivalents	High asynapsis in proximal regions of quadrivalents
**Refs**	Solari and Moses 1977	Codina-Pascual et al. 2006	Schmidt 2017	Virabyan et al. 2025	Matveevsky et al. 2017	this study	this study	this study	this study

Note: * - Interpretation by the authors of articles; (>diploid but < 2x) - above diploid, below tetraploid; ImmunoCyto – immunocytochemistry; stM-FISH - subtelomere-specific multiplex fluorescent-FISH; Chr – chromosome; NE - nuclear envelope; 2n – diploid number.

This study provides rare cytological evidence for polyploid meiocytes in four rodent species and reveals consistent anomalies during prophase I. The most prominent feature across all species was disturbed synapsis, particularly in the sex chromosomes, which likely disrupts progression through meiotic checkpoints and prevents the production of viable diploid sperm.

### Synapsis substages in quadrivalent configurations

**Figure 4.**
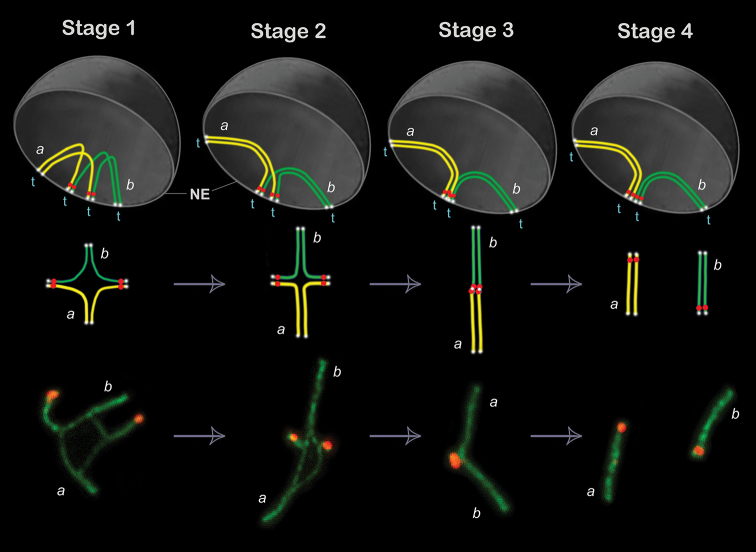
Synaptic adjustment and multivalent resolution: modelling the transition from quadrivalents to bivalents during prophase I progression in cases of sporadic meiotic tetraploidy. The model is based on the empirical data from the present and previous (Matveevsky et al. 2017) studies. Top row – simulation of quadrivalents within meiotic nuclei. Middle row – schematic representation of quadrivalent dynamics. Bottom row – examples of quadrivalent and bivalent configurations derived from a single cell (immunocytochemistry: axial and lateral elements of SCs are shown in green, centromeres in red). Abbreviations: NE – nuclear envelope; t – telomeres attached to the nuclear envelope (represented as white dots at chromosome ends in the top and middle panels). Color coding: a – yellow homologs; b – green homologs; red dots correspond to centromeres. Stage 1: Partial synapsis of four homologs (2 yellow, 2 green) forms a quadrivalent with sometimes broad asynaptic central region; configurations are often symmetrical. Stage 2: Synapsis progresses between homologous arms, reducing synaptic interactions between yellow and green homologs. Stage 3: Loss of synapsis between yellow and green homologs results in an extended, sometimes slightly curved, linear-like SC, resembling a bivalent, but unresolved (a pseudobivalent). Stage 4: Complete resolution of the quadrivalent into two bivalents.

Our observations, both current and previously published (Matveevsky et al. 2017), suggest a dynamic sequence of transitions from quadrivalents to individual bivalents, involving a series of distinct stages. These transitions exemplify an adaptive synaptic strategy that enables sporadic tetraploid cells to restore diploid-like pairing configurations and preserve the ability to progress through meiosis. The dynamics of quadrivalent resolution during meiotic prophase I in sporadic tetraploid cells can be summarized through four distinct stages (Fig. 4).

Stage 1 was designated ‘Quadrivalent assembly through partial synapsis of four homologs’. This initial stage is characterized by partial synapsis among four homologous chromosomes (two yellow and two green in the model of nucleus, Fig. 4) that leads to the establishment of a quadrivalent. Synapsis generally occurs along the arms of homologous chromosomes, frequently producing a symmetrical configuration. The hallmark of this stage is the presence of an extensive asynaptic region, reflecting areas of misaligned and/or incomplete pairing, near the center of a quadrivalent. This apparent symmetry may reflect a balance of mechanical forces provided by the telomeric ends, which remain tethered to the nuclear envelope. Here, we demonstrate for the first time that quadrivalent chromosomes assemble fully formed shelterin complexes at telomeric ends, as in *E. talpinus*. These telomeres are connected to the nuclear envelope through the LINC (SUN–KASH) complex, as in *E. alaicus*, similarly to normal spermatocytes.

Stage 2 was designated ‘Synaptic refinement and reconfiguration of a quadrivalent’. As meiosis progresses, synapsis becomes increasingly restricted to true homologous arms (i.e., yellow-yellow and green-green in Fig. 4), while synaptic interactions between heterologous homologs (yellow-green in Fig. 4) diminish. This results in a quadrivalent with two long and two short synapsed arms.

We designated stage 3 as ‘Transitional linearization of a quadrivalent into a pseudobivalent’. At this stage, synapsis between heterologous homologs is almost entirely lost, giving rise to a long, continuous SC structure. This SC is often linear and can exhibit mild central curvature, likely due to telomere-nuclear envelope attachments. Despite its visual resemblance to a bivalent, this structure retains features of a multivalent origin and has not undergone full resolution. We therefore designate it as a pseudobivalent.

Stage 4 is ‘Final quadrivalent resolution and formation of bivalents’. During the final stage, the quadrivalent structure fully resolves into two discrete bivalents, each of them is formed by homologous chromosomes of the original pairs (yellow-yellow and green-green in Fig. 4). The resulting bivalents are cytologically indistinguishable from those formed in normal meiocytes, indicating the successful correction of an initially aberrant tetraploid synaptic configuration.

Polyploid cells identified at the pachytene stage most likely originated from errors during premeiotic mitoses. Although some of these spermatocytes resemble syncytial fusions (Fig. 3, Suppl. material 1: figs S3, S4), others, like the cells in *E. tancrei* and *E. talpinus*, appear to be genuine tetraploids (Figs 1, 2, Suppl. material 1: figs S1, S2).

### Meiotic silencing in tetraploid spermatocytes

The presence of unsynapsed chromosomal regions during meiosis activates meiotic silencing of unsynapsed chromatin (MSUC), a transcriptional inactivation response that can affect genes essential for normal meiotic progression (Turner et al. 2005). MSUC is mediated by the recruitment of regulatory proteins and characteristic histone modifications to asynaptic chromosomal domains (Baarends et al. 2005). Mechanistically, MSUC is closely related to meiotic sex chromosome inactivation (MSCI), which primarily targets heteromorphic sex chromosomes or associated chromatin during male meiosis (Baarends et al. 2005; Schimenti 2005). The persistence of asynaptic regions within quadrivalents of tetraploid spermatocytes raises the question of their involvement in MSUC. We analyzed the localization of γH2AFX, a phosphorylated histone variant that initially marks meiotic DNA double-strand breaks and subsequently accumulates on chromosomal regions that fail to synapse, in *E. tancrei* and *E. talpinus*. Because γH2AFX is enriched on unsynapsed chromatin during both MSUC and MSCI, it is a well-established marker of these silencing responses (Baarends et al. 2005; Turner et al. 2005).

In *Ellobius tancrei* spermatocytes, identification of the XX sex chromosomes by γH2AFX staining revealed four unsynapsed axes, which remained centrally located within the nucleus rather than relocating to the nuclear periphery, as is typically observed in normal spermatocytes (Fig. 1). These axes, corresponding to univalents, were consistently γH2AFX-positive that indicate asynapsis. In contrast, asynaptic regions within quadrivalents lacked γH2AFX signals. It suggests that these chromosome segments could have undergone rapid synapsis followed by desynapsis, thereby, potentially escaping activation of the meiotic silencing mechanisms (Burgoyne et al. 2009). A somewhat different pattern was observed in *E. talpinus*, where some quadrivalents were entirely coated with γH2AFX, others lacked γH2AFX signals altogether, and still others exhibited a mosaic of γH2AFX-positive and γH2AFX-negative regions. Chromosomal domains devoid of γH2AFX are presumed to have undergone a brief synaptic phase followed by desynapsis, whereas γH2AFX-positive regions likely remain unsynapsed. This temporal desynchronization of synapsis may reflect genomic instability of tetraploid meiotic cells.

Incomplete synapsis (asynaptic regions) and improper recombination are well-known triggers of meiotic arrest (Turner et al. 2005; Subramanian and Hochwagen 2014). Our findings support the idea that disrupted synapsis and meiotic sex chromosome inactivation (MSCI) failure in tetraploid cells can activate checkpoint pathways, leading to meiotic arrest and likely elimination (Li et al. 2009). Rare escape from these checkpoints could explain the exceptionally low frequency of diploid spermatozoa in humans: from 0.0001% to 0.34% in different studies (Lu et al. 1994; Rodrigo et al. 2010). A clinically relevant example of such rare checkpoint escape is macrozoospermia, or sperm macrocephaly syndrome (SMS), a rare cause of male infertility (<1%), most commonly associated with homozygous mutations in the aurora kinase C gene, which disrupts the second meiotic division and lead to chromosome doubling and formation of large-headed, often polyploid spermatozoa (Perrin et al. 2008; Ben Khelifa et al. 2011). These cases suggest that defective meiotic divisions and incomplete checkpoint control, consistent with the cytological abnormalities observed here, can occasionally permit progression of polyploid germ cells into spermiogenesis (Fellmeth et al. 2016; Carmignac et al. 2017).

Although rare polyploid embryos occur, they are usually mosaics with severe abnormalities and early mortality (Cavalcanti, Zanchetta 2005; Schacht et al. 2018). Stable germline transmission of polyploid genomes in mammals remains virtually nonexistent.

## Conclusion

The rare occurrence of polyploid spermatocytes in four studied rodent species highlights polyploidy as a notable deviation in mammalian meiosis. Several key findings were revealed in synaptic configuration, recombination events, and transcriptional inactivation of meiotic chromatin. Quadrivalents in tetraploid spermatocytes generally displayed symmetric synapsis in distal regions and asynapsis proximally. Some quadrivalents resembled adjacent bivalents connected at telomeric ends, suggesting partial resolution of quadrivalents; this process likely assisted by SUN1-mediated nuclear envelope anchoring (as observed in *E. alaicus*). Despite asynapsis, recombination foci (MLH1) were present in many quadrivalents and bivalents, albeit with altered positioning (as observed in *E. tancrei*). This indicates that some homolog interactions and crossover formation remain functional, though possibly desynchronized or spatially misregulated. Over-diploid cells consistently display disrupted synapsis for sex chromosomes. Such abnormalities likely trigger early meiotic checkpoints and lead to cellular elimination before spermiogenesis.

Although tetraploid cells may occasionally proceed further through meiosis, the resulting spermatozoa are typically non-viable or eliminated, and their participation in fertilization is exceedingly rare. Cases of over-diploid embryos in humans are associated with severe developmental abnormalities and do not support heritable polyploidy.

Our findings reinforce the hypothesis that mammalian sex chromosomes, through mechanisms such as MSCI, serve as evolutionary safeguards against polyploid genome inheritance. So, polyploidy in mammalian gametogenesis is a genetic anomaly rather than an adaptive mechanism. Meiotic checkpoints and nuclear architecture prevent the inheritance of such overcomplete genomes, making polyploid evolution highly improbable in mammals, unlike in plants or lower vertebrates.

## Authors’ contributions

Conceptualization: S.M., O.K. and I.B.; methodology: O.K. and S.M.; investigation: S.M., T.G., V.T., A.B., and I.B.; writing: S.M., V.T., A.B., I.B. and O.K.; visualization: S.M. and I.B. All authors have read and agreed to the published version of the manuscript.

## Supplementary materials

Supplementary material 1 Additional information Data type docx Explanation note **fig. S1**. Tetraploid spermatocytes of the *E. tancrei* (A, see Fig. 1), *E. talpinus* (B, see Fig. 2) and *N. leucodon* (C, see Fig. 3). **fig. S2**. Tetraploid spermatocyte of the *E. tancrei* specimen # 25187, 2n = 49, 1Rb2.11, 1Rb2.18, 2Rb5.9, 1Rb3.18, the diplotene stage. **fig. S3**. Various configurations of chromosomal quadrivalents in tetraploid spermatocytes of the Alay mole vole *E. alaicus* (A–H). **fig. S4**. Two tetraploid and several diploid spermatocytes of the Alay mole vole *E. alaicus*, the pachytene stage. **fig. S5**. A zygotene spermatocyte of the common mole vole *E. talpinus*, #27041 (A, B). https://binary.pensoft.net/file/1518238This dataset is made available under the Open Database License (http://opendatacommons.org/licenses/odbl/1.0/). The Open Database License (ODbL) is a license agreement intended to allow users to freely share, modify, and use this Dataset while maintaining this same freedom for others, provided that the original source and author(s) are credited. Sergey Matveevsky, Oxana Kolomiets, Tatiana Grishaeva, Aleksey Bogdanov, Valentina Tambovtseva, Irina Bakloushinskaya
