# Supplementary material for: The forbidden doubling: exploring rare spermatocyte polyploidy in mammals
Source: Comp Cytogenet. 2026 Jan 23;20:67–85. doi: 10.3897/compcytogen.20.177662 (PMC12859646; doi:10.3897/compcytogen.20.177662)
Supplement: Supplementary material 1 — Additional information [file comparative_cytogenetics-20-067_article-177662__-s001.docx]

Supplementary materials

**The forbidden doubling: exploring rare spermatocyte polyploidy in mammals**

Sergey Matveevsky^1^, Oxana Kolomiets^1,^*, Tatiana Grishaeva^1^, Aleksey Bogdanov^2^, Valentina Tambovtseva^2^ and Irina Bakloushinskaya^2^

^1^ Vavilov Institute of General Genetics, Russian Academy of Sciences, Moscow 119991, Russia

^2^ Koltzov Institute of Developmental Biology, Russian Academy of Sciences, Moscow 119334, Russia

Corresponding author: S. Matveevsky (e-mail: sergey8585@mail.ru)

* Professor Oxana Kolomiets died prior to the submission of this paper.


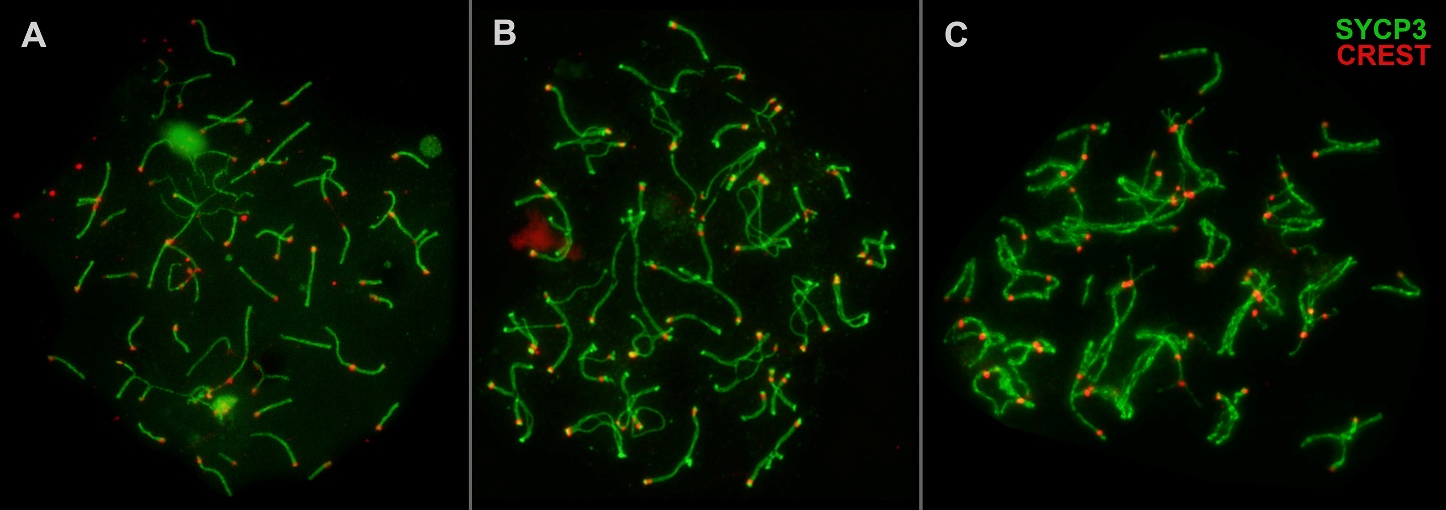


**Figure S1.** Tetraploid spermatocytes of the *E. tancrei* (A, see Fig. 1)*, E. talpinus* (B, see Fig. 2) *and N. leucodon* (C, see Fig. 3)*.*

**
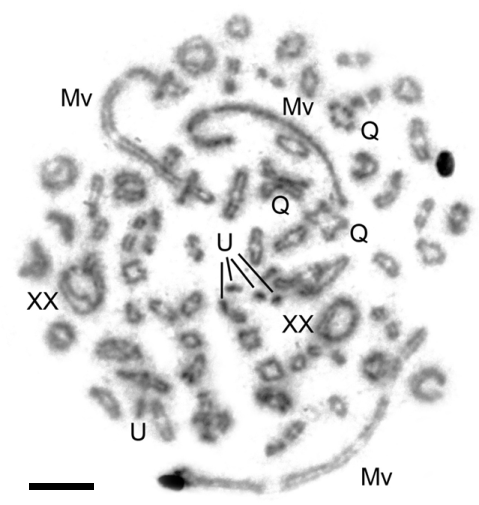
**

**Figure S2.** Tetraploid spermatocyte of the *E. tancrei* specimen # 25187, 2n=49, 1Rb2.11, 1Rb2.18, 2Rb5.9, 1Rb3.18, the diplotene stage. Quadrivalents were formed by homologous acrocentrics (Q), multivalents were originated due to heterozygosity for Robertsonian translocations (Mv), univalents are marked as U, two sex bivalents are marked as XX. Scale bar = 5 µm.


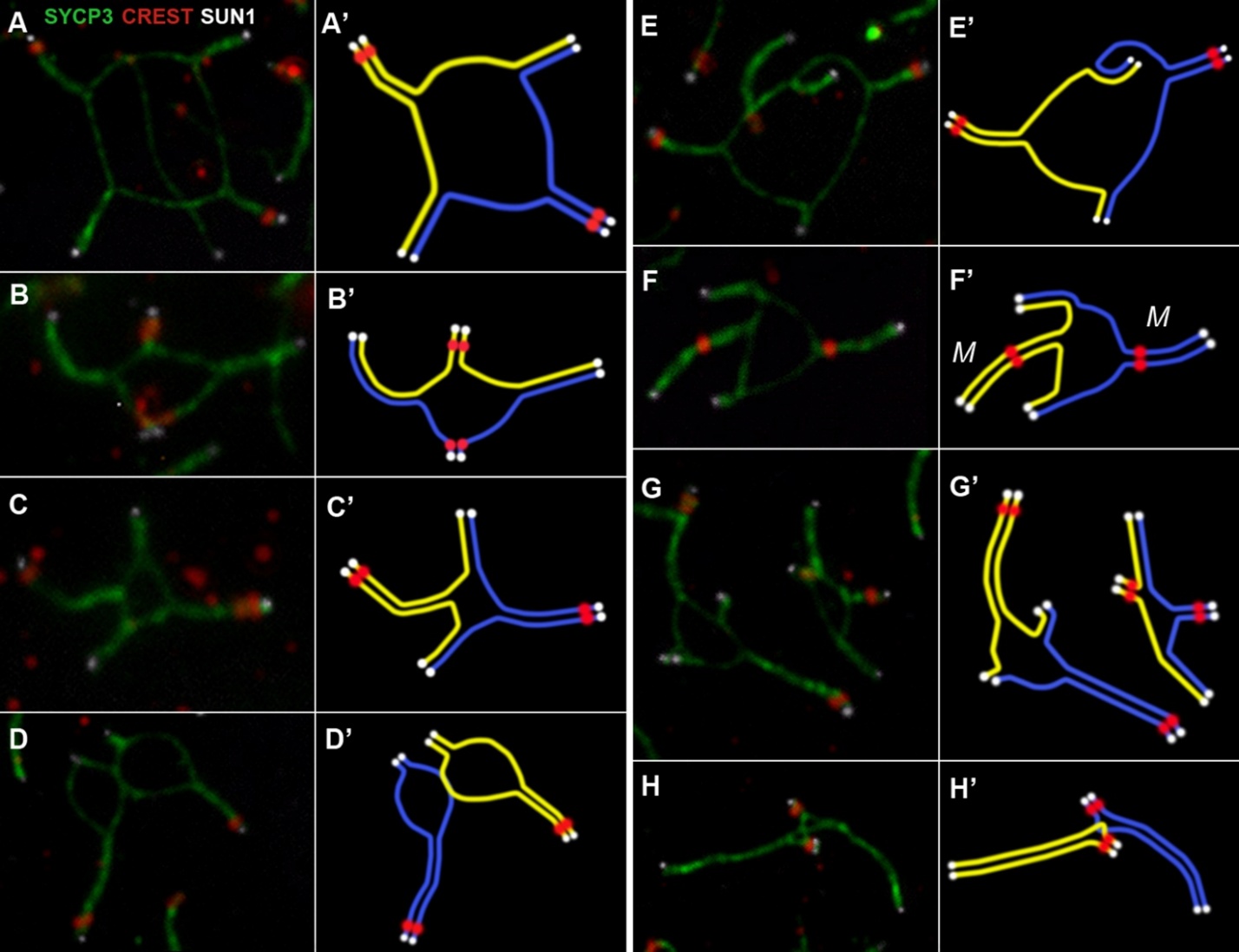


**Figure S3.** Various configurations of chromosomal quadrivalents in tetraploid spermatocytes of the Alay mole vole *E. alaicus* (A-H). Axial and lateral elements of SCs were identified using an anti-SYCP3 antibody (green). Centromeres were identified using "CREST"—antibodies to kinetochore proteins (red). LINСs in the nuclear envelope were identified using SUN1 antibodies (white). All quadrivalents were taken from two meiotic nuclei presented in Supplementary Fig. 3. Each quadrivalent has an interpretation scheme (A’-H’). In panels F', 'M' labels metacentric chromosome configurations.


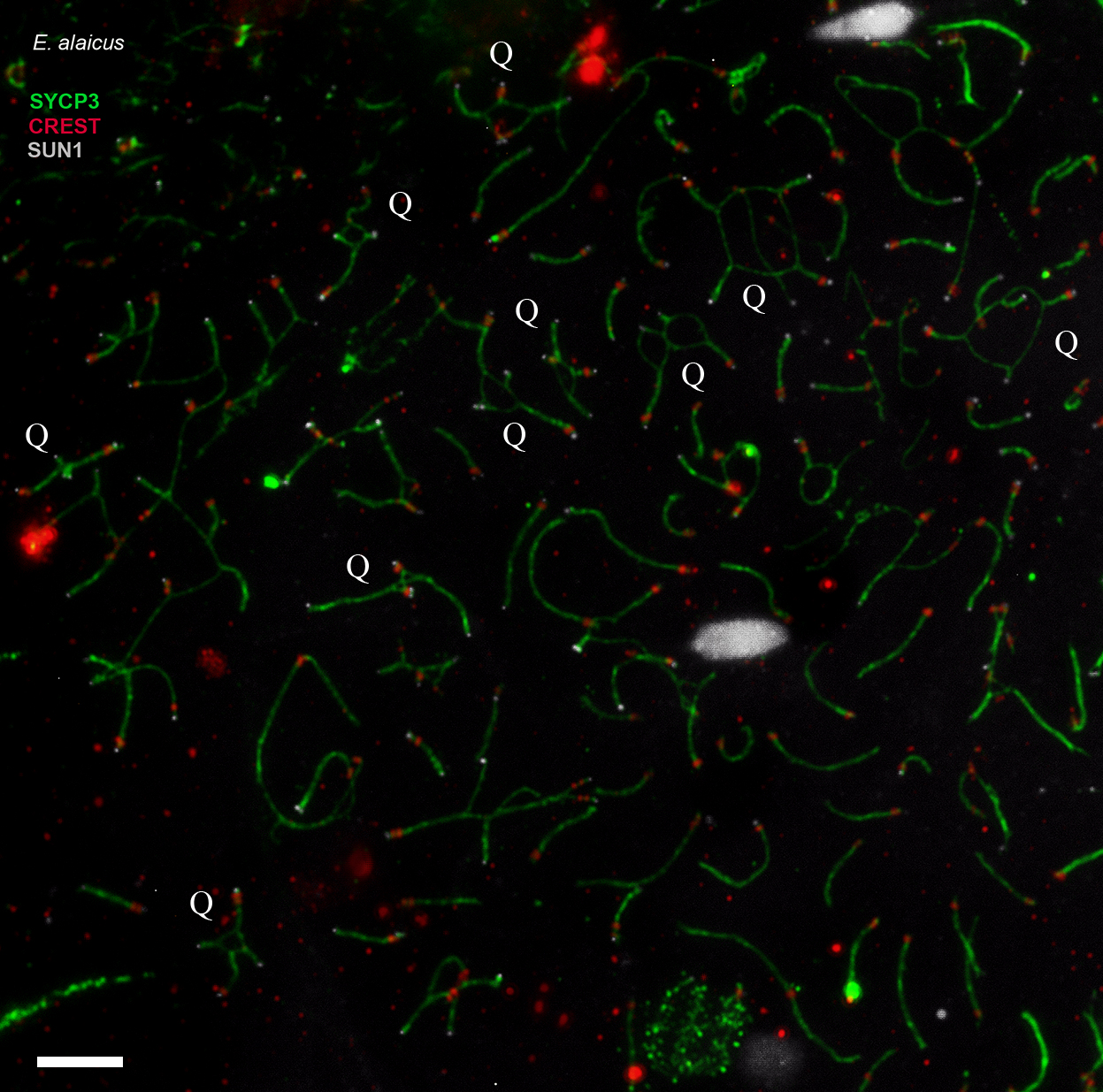


**Figure S4.** Two tetraploid and several diploid spermatocytes of the Alay mole vole *E. alaicus,* the pachytene stage. Axial and lateral elements of SCs were identified using an anti-SYCP3 antibody (green). Centromeres were identified using "CREST"—antibodies to kinetochore proteins (red). LINСs in the nuclear envelope were identified using SUN1 antibodies (white). Q – chromosomal quadrivalent. Scale bar = 5 µm.


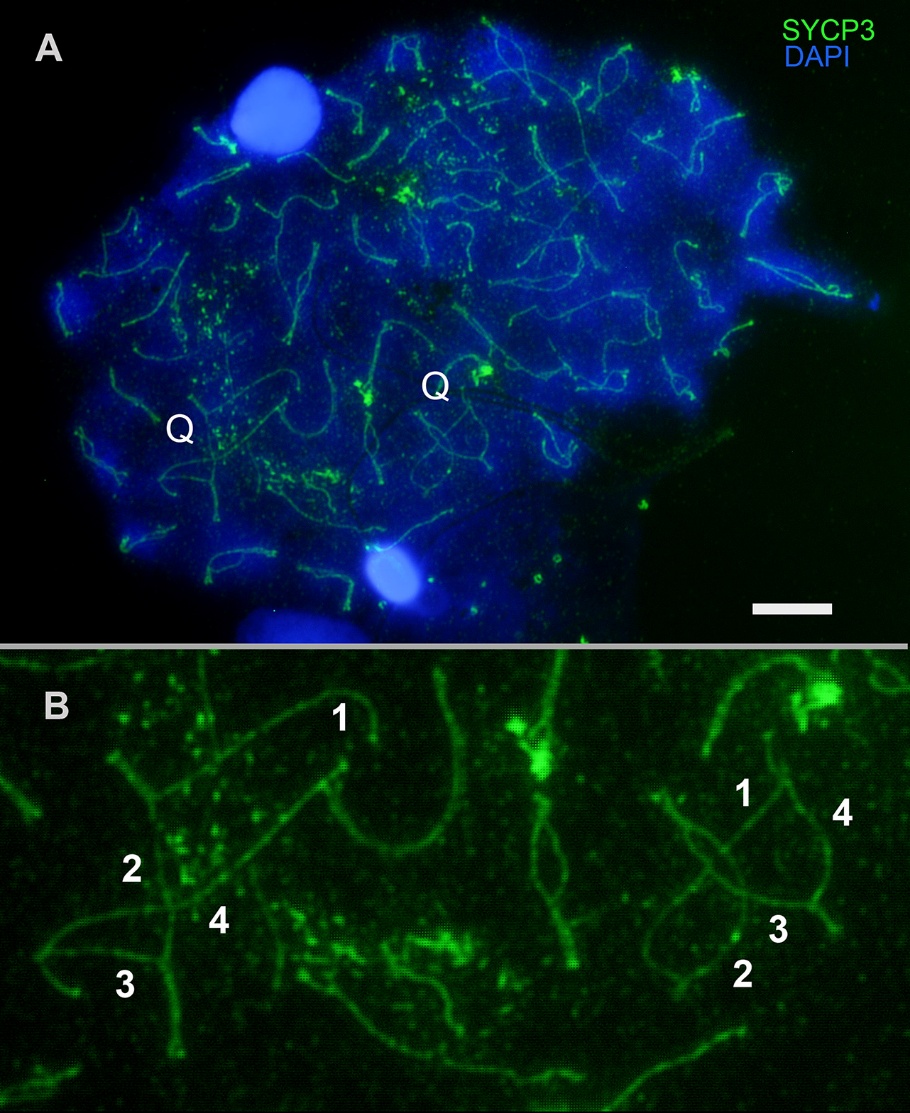


**Figure S5.** A zygotene spermatocyte of the common mole vole *E. talpinus*, #27041 (A-B). Axial and lateral elements of the SCs were identified using an anti-SYCP3 antibody (green). Chromatin was stained with DAPI (blue). The lower cell exhibits quadrivalent-like (Q) configurations, more visible in enlarged fragment (B) of microscopic image A. Scale bar = 5 µm.
